# *Kigelia africana* fruit fractions inhibit in vitro alpha-glucosidase activity: a potential natural alpha-glucosidase inhibitor

**DOI:** 10.1186/s12906-024-04510-5

**Published:** 2024-06-12

**Authors:** Tumelo Akapelwa Muyenga, Samuel K. Dominion Bamitale, Dan Kibuule, Simbarashe Sithole, Stanley Mukanganyama, Carlen Rudolph, Luanne Venables, Anna C. Hattingh, Maryna van de Venter, Christian Chinyere Ezeala

**Affiliations:** 1 Department of Physiological Sciences, School of Medicine and Health Sciences, Mulungushi University, P.O. Box 60009, Livingstone, Zambia; 2 Department of Pharmacology and Therapeutics, Faculty of Health and Veterinary Sciences, University of Namibia, Windhoek, Namibia; 3 Department of Internal Medicine and Pharmacology, Faculty of Medicine, and Health Sciences, Eastern Cape, Walter Sisulu University, Mthatha, South Africa; 4 Department of Pharmacology and Therapeutics, Faculty of Health Sciences, Busitema University, Busitema, Uganda; 5 Department of Chemistry and Earth Sciences, University of Zimbabwe, Mt. Pleasant, Harare, Zimbabwe; 6 Department of Biotechnology and Biotechnology, University of Zimbabwe, Mt. Pleasant, Harare, Zimbabwe; 7 Department of Biochemistry and Microbiology, Nelson Mandela University, Port Elizabeth, South Africa; 8 College of Health Agriculture and Natural Sciences, Africa University, Mutare, Zimbabwe

**Keywords:** Alpha-amylase inhibition, Alpha-glucosidase inhibition, *Kigelia africana* fruit fractions, Gas Chromatography- mass spectrometry, Diabetes, Phytochemistry

## Abstract

**Background:**

Diabetes affects 75% of people in low-income countries, where conventional drugs like metformin are available, but newer drugs like alpha-glucosidase inhibitors are not accessible to most Southern African patients.

**Aim:**

To evaluate the α-glucosidase and α-amylase inhibitory activities of fractionated aqueous extracts of *Kigelia africana* fruit (KAFE) and their phytochemical fingerprints using gas chromatography-mass spectrometry (GC–MS).

**Materials and methods:**

We studied *K. africana* fruit fractions' inhibitory effects on alpha-glucosidase and alpha-amylase using bioassay-guided fractionation, and analyzed their phytochemical profiles with GC–MS.

**Key findings:**

Both the aqueous extract and ethyl acetate fraction of the aqueous extract exhibited a low dose-dependent inhibition of alpha-amylase activity (p < 0.0001). At a concentration of 500 μg/mL, the aqueous extract caused an alpha-glucosidase inhibition of 64.10 ± 2.7%, with an estimated IC50 of 193.7 μg/mL, while the ethyl acetate fraction had an inhibition of 89.82 ± 0.8% and an estimated IC50 of 10.41 μg/mL. The subfraction G, which had the highest alpha-glucosidase inhibitory activity at 85.10 ± 0.7%, had significantly lower activity than the ethyl acetate fraction. The most bioactive fraction was found to contain 11"(2-cyclopenten-1-yl) undecanoic acid, ( +)- and cyclopentane undecanoic acid as well as the indole alkaloids Akuammilan-17-ol-10-methoxy, N-nitroso-2-methyl-oxazolidine and epoxide Oxirane2.2″ -(1.4-butanediyl) bis-.

**Conclusion:**

The *K. africana* fruit fraction demonstrated significant alpha-glucosidase inhibitory activity, while its alpha-amylase inhibitory activity was limited. This study suggests a potential natural alpha-glucosidase inhibitor and phytocompounds that could serve as leads for developing antidiabetic agents.

## Highlights of the study

1. The study highlights the *in vitro* alpha-glucosidase inhibitory activity of *Kigelia* fruit extract and its fractions with estimated IC_50_ of aqueous crude extract at 193.7 μg/mL and ethyl acetate fraction 10.41μg/mL.

2. The study has highlighted rare cyclic fatty acids 11"(2-cyclopenten-1-yl) undecanoic acid, (+)- and cyclopentane undecanoic acid; indole alkaloids akuammilan-17-ol- 10-methoxy, N-nitroso-2-methyl-oxazolidine and epoxide oxirane2.2” -(1.4-butanediyl) bis- and a naturally occurring phthalic acid ester in the bioactive subfractions.

## Introduction

The burden of diabetes mellitus is substantial in low-income countries, with three of the four individuals with diabetes residing in low- and middle-income countries [1, 2]. In Zambia, it is estimated that 726,300 adults live with diabetes [2]. A significant number of these patients also experience complications such as vision impairment, sexual dysfunction, and fatigue, as well as comorbidities such as obesity, stroke, and hypertension [3, 4]. While conventional drugs such as metformin are available for management, recent drug groups such as alpha-glucosidase inhibitors that may be used effectively in prediabetes and to alleviate metabolic syndrome of diabetes are not readily available to most Southern African patients (Rossiter, 2014). This warrants the need to investigate the possible sources of these drug groups to provide a local solution. Furthermore, in cases where alpha-glucosidase inhibitors are available, these drugs are not devoid of adverse drug reactions [5]. Therefore, there is a need to find more alpha-glucosidase inhibitors with fewer adverse drug reactions.

*Kigelia africana (Lam) Benth. (Mupolota in Silozi or Muzungula in Tonga)* is a medicinal plant that has attracted the attention of researchers because it is traditionally used to lower blood sugar levels [6]. Previous studies have shown that crude extracts from multiple parts of the plant (leaves, flowers, and fruits) can lower blood sugar levels in diabetes-induced mouse models [7, 8]. However, very few studies have highlighted the compounds responsible for the observed antidiabetic activity and bioactivity of the fruit extracts concerning their blood glucose-lowering effect. Although studies have determined the basic phytochemistry of *Kigelia* concerning diabetes, it has often been associated with the presence of alkaloids, iridoids, and phenolic compounds [9–11]. Furthermore, while some studies have demonstrated the plant extracts’ ability to enhance glucose utilization in muscle, as well as improve insulin secretion [12], there is scanty information that discusses the postprandial benefits of Kigelia extracts. Literature has also highlighted plants that are potential alpha-glucosidase inhibitors [13, 14].

Thus, in this study, through active fractionats we aimed to determine the alpha-glucosidase and alpha-amylase inhibitory activity of the fractionated aqueous extracts of *Kigelia africana* fruit (KAFE). Additionally, we aimed to associate their phytochemical fingerprints using GC–MS to the observed bioactivity.

## Materials and methods

### Chemicals and reagents

The materials utilized in this study consisted of various analytical standard substances obtained from Merck, South Africa., namely; hexane, chloroform, ethyl acetate, methanol, porcine pancreatin buffer, potassium monobasic anhydrous phosphate, reduced glutathione, p-NP-Gluc, p-Nitrophenyl α-D-glucopyranoside, Na_2_CO_3_ sodium carbonate, α–glucosidase from Saccharomyces cerevisiae, Positive control for alpha-glucosidase assay: Epigallocatechin gallate (EGCG), Silica gel.

### Plant material collection and authentication

The *K. africa*na fruit was collected with permission in January 2022, in the Kazungula District specifically from the riverine area of Singanga village in Kachola, Chief Sekute area, in the Southern Province of Zambia. The plant material underwent authentication in the Department of Biological Sciences within the School of Natural Sciences at the University of Zambia (UNZA), by Florence Nyirenda (MSc) a scientist. The specimen had receipt number 1793751 and was mounted in the herbarium under specimen number 22, 420.

### Plant extraction and fractionation

The fruits of *K. africana* were subjected to cutting, mincing, and subsequent air drying within a designated shed for one week. The fruits were subsequently pulverized and filtered using a screen with a hole diameter of 0.6 mm to acquire a uniform powder, which was stored at a temperature of 10 °C. A quantity of 500 g of powdered *K. africana* fruit was utilized in a Soxhlet extraction process, employing 2500 mL of water as the extracting solvent, resulting in the production of an aqueous extract. The extracted sample was subjected to a drying process under decreased pressure utilizing a Rotavapor at a temperature of 45 °C. Subsequently, a liquid–liquid fractionation technique was employed to separate the extract.

About 50 g of ethyl acetate fraction was mixed with 50 g of silica gel, then subjected to the column fraction, using a glass column of diameter 200 mm and 2 m length. The column was packed with 400 g of silica while 1.330 L of hexane was used to form a slurry. Once the sample had settled, the hexane was collected in a conical flask while ensuring that the column did not dry out the sample at the top. The following solvent systems were used to obtain fractions of hexane/ethyl acetate (100%; 97.5:2.5; through to 10:90) and then Ethyl acetate/methanol (100%; 95:5 through to 90:10). Guided by TLC, bands with similar mobility were grouped and a total of thirteen fractions were collected.

### In vitro* postprandial activity*

The collected fractions were tested for *in vitro* inhibition of alpha-glucosidase and alpha-amylase enzymes according to the methodology given by Pingle et al*.* (2021).

### Test sample preparation

Test samples were reconstituted in dimethyl sulfoxide (DMSO) to a final concentration of 100 mg/mL. Samples were sonicated if insoluble and stored at 4 °C until required. Samples were diluted in each respective assay buffer to concentrations as specified.

### Alpha amylase inhibitory activity

The method of Xiao et al. (2006), modified as described by Pringle et al. (2021) was used. In a 96-well microtiter plate, 15 μL of the test sample (aqueous crude extract, ethyl acetate fraction or acarbose as positive control) was incubated for 10 min at 37 °C with 5 μL of 1 mg/mL porcine pancreatin in phosphate-buffered saline. To initiate the reaction, 20 μL of 2 mg/mL starch solution was added followed by incubation for 30 min at 37 °C. The 2 mg/mL starch solution was prepared by boiling with continuous stirring for 15 min until the solution turned clear; it was then cooled to room temperature with continuous stirring and the volume of evaporated water was replaced. At the end of the 30-min incubation, the reaction was halted by adding 10 μL 1 M HCl and 75 μL iodine reagent (0.127 g iodine and 0.083 g potassium iodide in 100 mL distilled water). The absorbance was measured at 580 nm. Controls containing no enzyme or substrate were included for each sample to account for the absorbance of the extracts. The absorbance of the enzyme- and substrate-free wells was subtracted from the absorbance readings of the wells containing enzyme and substrate, and the percentage of α-amylase inhibition was calculated using the following formula:

$$\%\ \alpha\ amylase\text{ inhibition }= \frac{(\text{amylase activity of control}-\text{ amylase activity of test sample})}{\text{amylase activity of control}}\times100$$

where: amylase activity = A 580 nm without enzyme—A 580 nm with enzyme:

### Alpha-glucosidase activity

The method of Akinloye et al. (2012) as modified by Pringle et al. (2021) was used. In a 96-well plate, 15 μL of the sample (aqueous crude extract, ethyl acetate fraction or epigallocatechin gallate (ECGC) as positive control), 20 μL of 50 µg/mL enzyme and 60 μL reaction buffer (67 mM potassium phosphate, pH 6.8 to which 3 mM reduced glutathione was added directly before use) was pre-incubated for 5 min at 37 °C. Ten microlitres of 10 mM p-nitrophenyl α-D-glucopyranoside substrate was added, followed by incubation for 30 min at 37 °C. The reaction was halted by adding 25 μL of 100 mM Na_2_CO_3_. Controls containing no enzyme or substrate were included for each sample to account for the absorbance of the extracts. The amount of p-nitrophenol released was determined spectrophotometrically at 405 nm. The percentage of α-glucosidase inhibition was calculated as follows:

$${\%}\;\alpha-glucosidase\;\mathrm{inhibition}=\frac{\left(\mathrm A410\mathrm{nm}\;\mathrm{of}\;\mathrm{control}-\;\mathrm A410\mathrm{nm}\;\mathrm{of}\;\mathrm{test}\;\mathrm{sample}\right)}{\mathrm A410\mathrm{nm}\;\mathrm{of}\;\mathrm{control}}\times100$$

The absorbance of the enzyme and substrate-free wells was subtracted from the absorbance readings of the wells containing enzyme and substrate.

### GC–MS analysis

GCMS analysis of the bioactive subfractions was performed on the Scion 436 GC–MS Single Quadruple equipped with a low bleed, high inertness SCION 5MS column (equivalent to 5% phenyl/95% dimethyl polysiloxane). Capillary column (30 m × 250 m × 0.25 m). Pure helium gas (99.99%) with a constant flow rate of 1 ml/min was used as the carrier gas. For spectral detection, an electron ionization energy method was used with a high ionization energy of 70 eV (electron volts) with a scan time of 0.2 s and fragments in the range of 40 to 600 m/z. The injection quantity of 1µL was used with a split ratio of 10:1 and an injection temperature of 250 °C (constant). The column oven temperature was set at 50 °C for 3 min, increased at 10 °C per minute to 280 °C and the final temperature increased to 300 °C for 10 min. The phytochemicals present in the fractions were identified by comparing their retention time (min), peak area, peak height, and mass spectral pattern with the spectral database of authentic compounds stored in the National Institute of Standards and Technology (NIST) library.

## Results

### Alpha amylase inhibitory activity

**Fig. 1:**
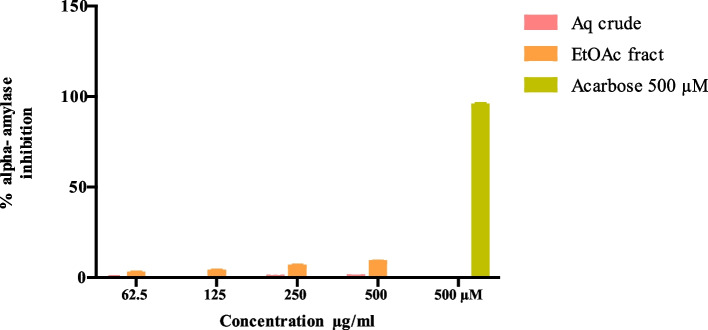
Percentage (%) inhibition of alpha-amylase by Aqueous extract and Ethyl acetate fraction. Data points determined via ANOVA represent the mean ± SD of quadruplicates. ** (*p* < 0.0001) compared to Acarbose 500µM

The aqueous crude extract did not display any alpha-amylase inhibitory activity. The ethyl acetate fraction of the aqueous extract displayed a very low dose-dependent alpha-amylase inhibitory activity. However, this activity was significantly less than that of the positive control, acarbose(*p* < 0.0001; Fig. 1).

### Alpha-glucosidase inhibitory activity

**Fig. 2:**
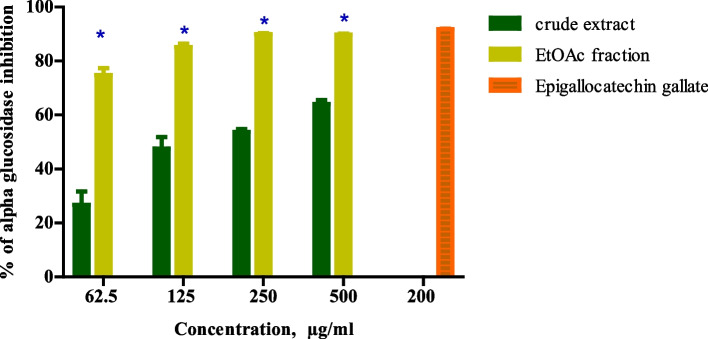
Percentage (%) inhibition of alpha-glucosidase by *Kigelia* aqueous crude extract and Ethyl acetate fraction (*) shows a significantly higher % inhibition of ethyl acetate fractions compared to Kigelia aqueous crude extracts at the same concentrations (*p* < 0.0001)

The aqueous extract showed a dose-dependent increase in the alpha-glucosidase inhibitory activity. At the highest concentration of 500 μg/mL, there was 64.10 ± 2.7% inhibition. In contrast, the study observed a statistically significant increase in the alpha-glucosidase inhibitory activity of the ethyl acetate fraction (*p* < 0.0001) compared to the crude extract. At 500 μg/mL, there was an 89.82 ± 0.8% inhibition of the fractions. However, this was statistically significantly lower than the % inhibition of the positive control which had 94.88 ± 0.1% inhibition at 200 μg/mL, see Fig. 2.

An estimated IC_50_ for the aqueous crude extract was determined to be IC_50_ – 193.7 μg/mL while that of ethyl acetate fraction was determined to be IC_50_ – 10.41 μg/mL.

**Fig. 3:**
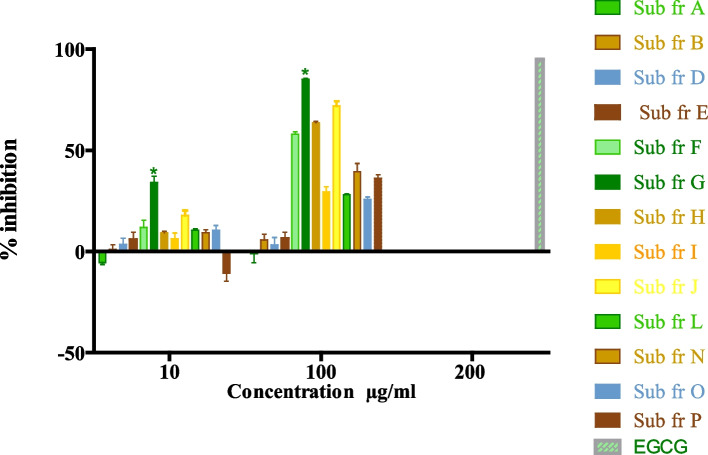
% inhibition of alpha-glucosidase enzyme by Kigelia subfractions *(*p* < 0.0001) statistically significant difference of % inhibition among fractions

Thirteen subfractions collected from the ethyl acetate fraction were tested for alpha-glucosidase inhibitory activity. Consequently, subfractions F, G, H and J had the best activity. Subfraction G had the highest inhibitory activity at 85.10 ± 0.7%. as shown in Fig. 3.

### GC–MS analysis of bioactive fractions

The GC–MS analysis of fractions F, G, H, and J showed the presence of fatty acids, phenolic compounds as well as nitrogen-containing compounds as indicated in Tables 1, 2, 3 and 4 below. The GC–MS analysis of subfractions F, G, H, and J had several peaks that were distributed among the sub-fractions.

**Table 1:**
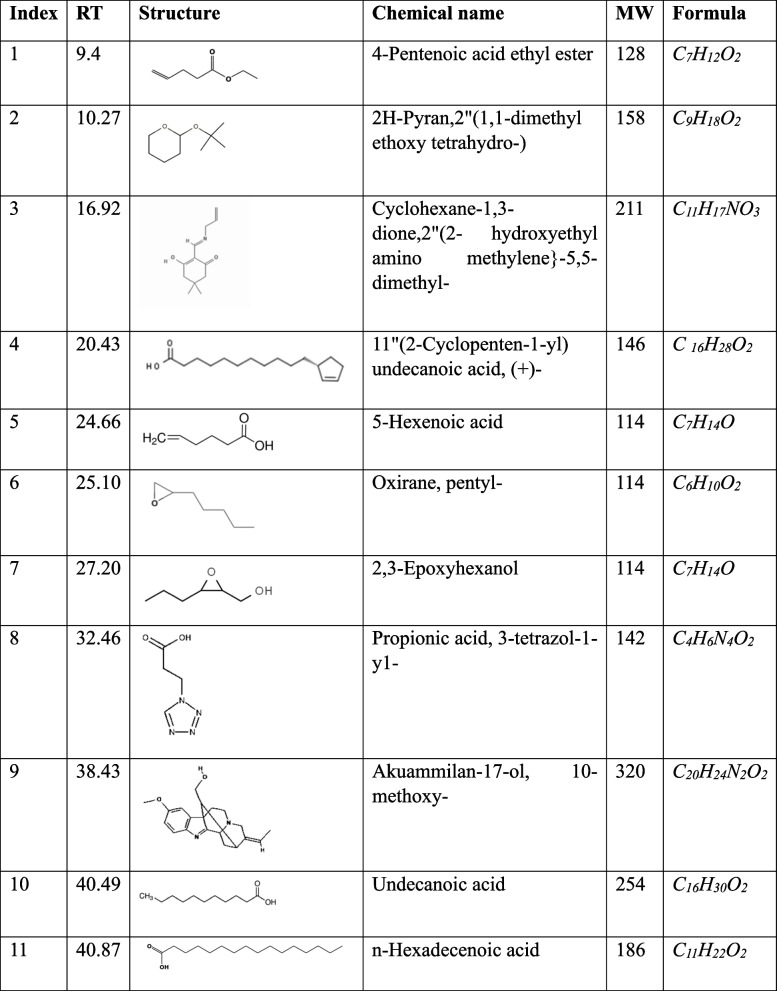
Subfraction F peaks

**Table 2:**
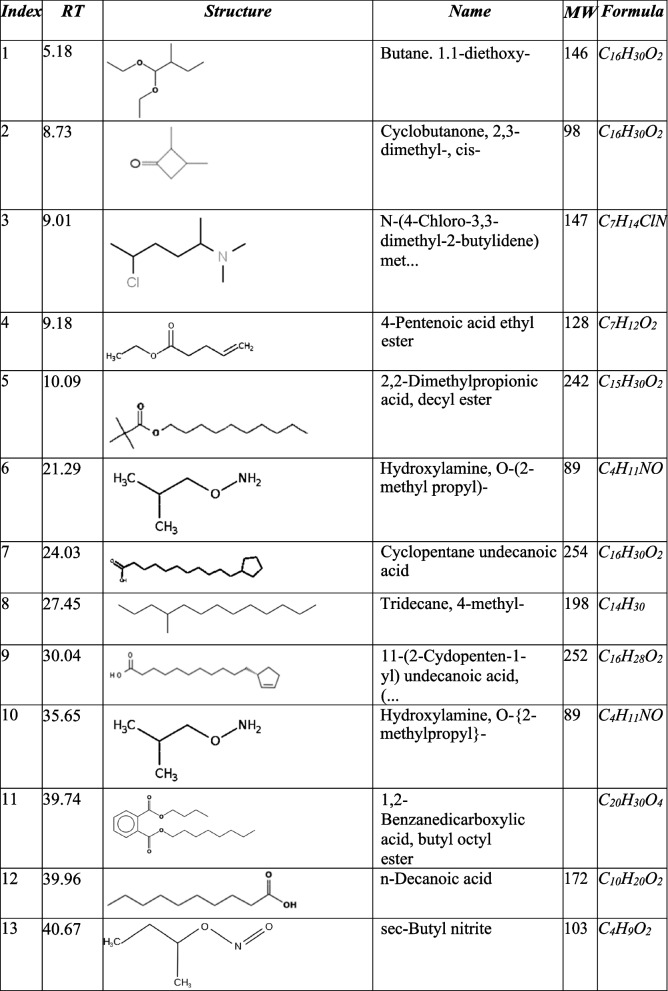
Subfraction G phytocompunds

**Table 3:**
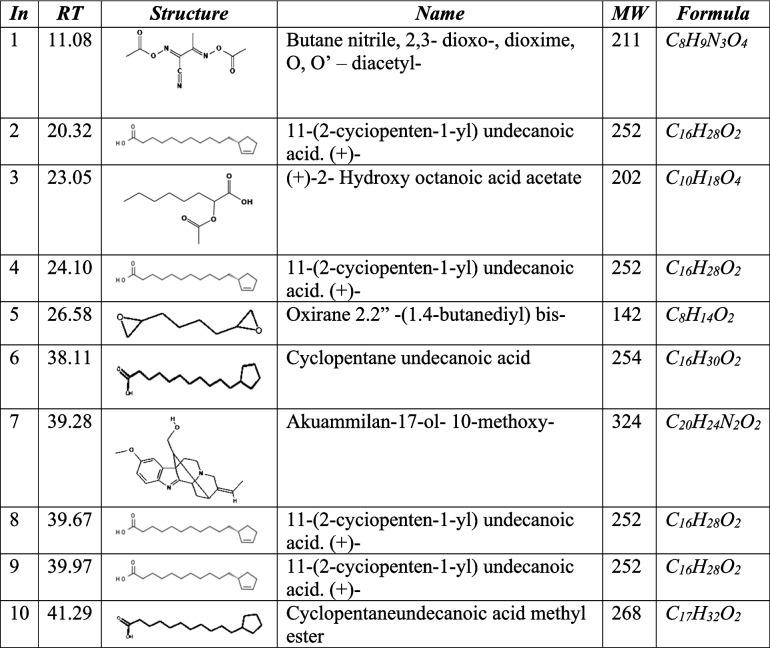
Subfraction H phytocompounds

**Table 4:**
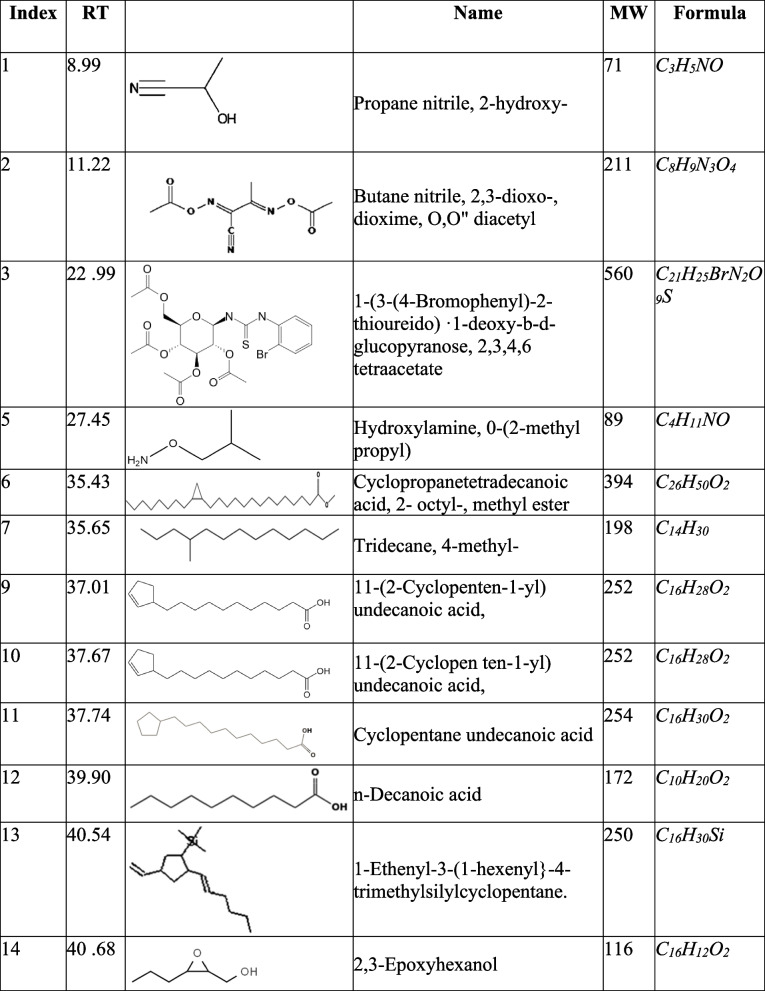
Subfraction J phytocompounds

### Subfraction F.

**Fig. 4:**
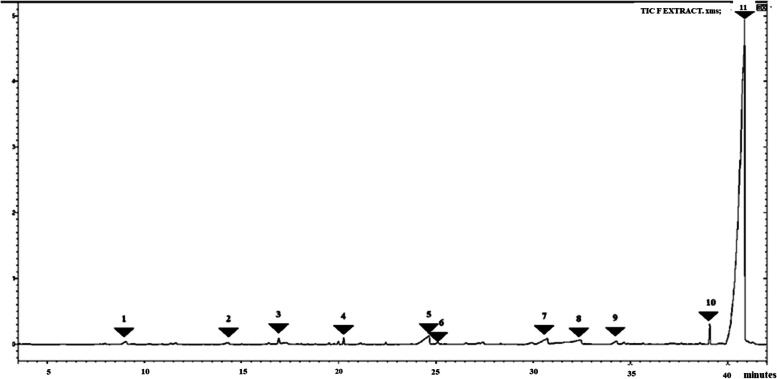
GC–MS chromatogram of subfraction F. The numbers indicated in the spectrogram correspond with the peaks of compounds indicated in Table 1 below

Subfraction F had the largest number of peaks. However, there were 11 peaks of interest which have been indicated in Fig. 4 and Table 1 below the highest peak corresponded to n-hexadecenoic acid, followed by undecanoic acid at 40.8 and 40.4 min, respectively.

### Subfraction G

**Fig. 5:**
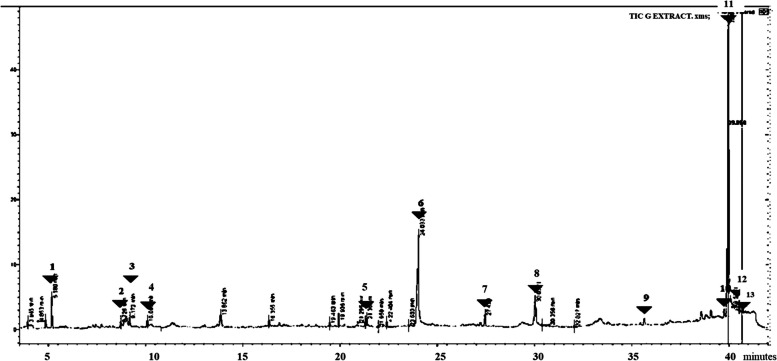
GC–MS chromatogram for sub-fraction G. The numbers indicated in the spectrogram correspond with the peaks of compounds indicated in Table 2 below

Under subfraction G, we recorded 13 peaks with the highest peak corresponding to n-decanoic acid at 39.95 min. At 30.06 min, the peak corresponded to 11"(2-cyclopenten-1-yl) undecanoic acid, ( +)-. Other peaks of interest at 24.08 and 27.4 min corresponded to cyclopentane undecanoic acid and tridecane, 4-methyl- respectively. The highest peak corresponded to 1,2-benzene dicarboxylic acid and butyl octyl ester. Data is shown in Fig. 5 and Table 2.

### Subfraction H

**Fig. 6:**
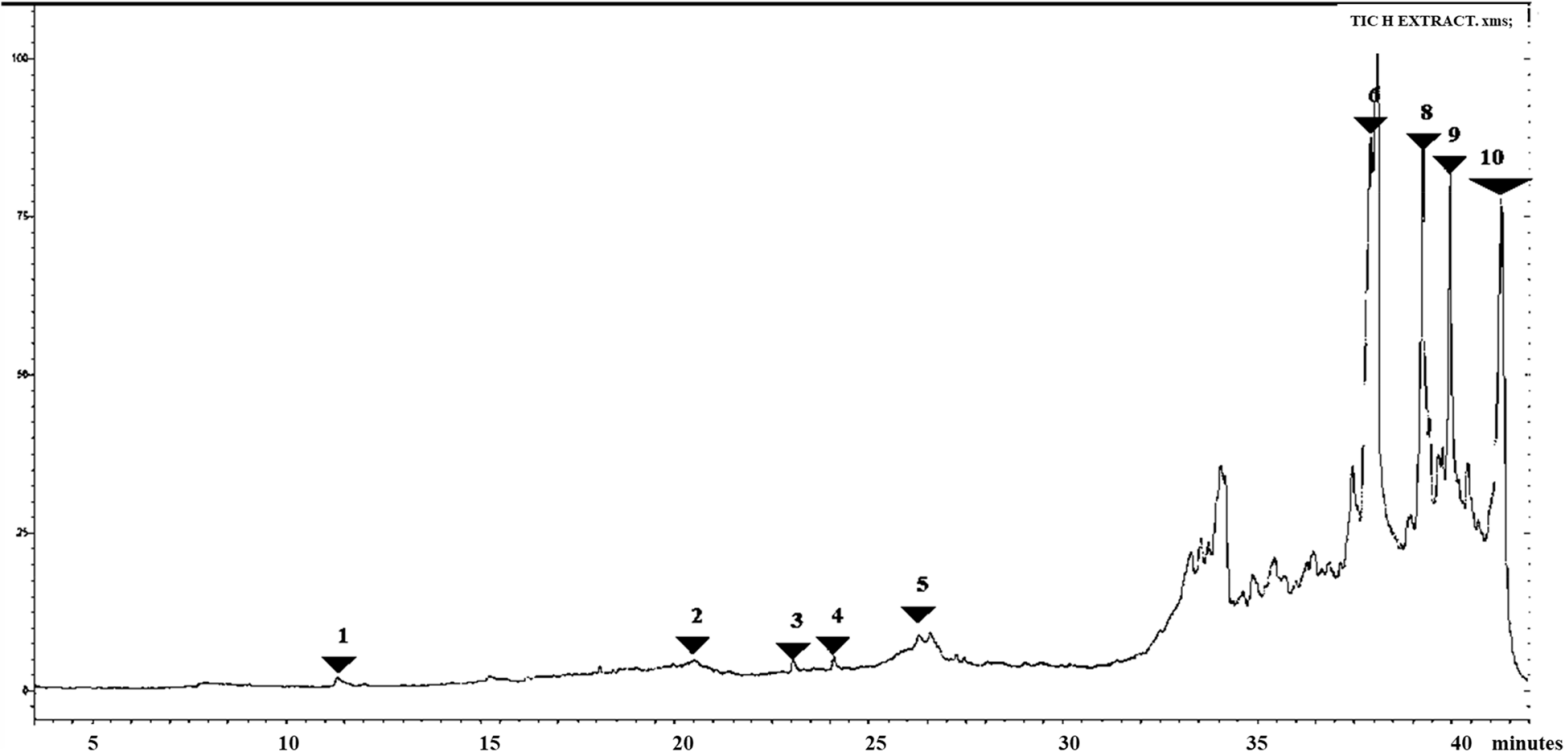
GC–MS chromatogram for sub-fraction H. The numbers indicated in the spectrogram correspond with the peaks of compounds indicated in Table 3 below

Subfraction H had 30 peaks, most of which corresponded to 11"(2-cyclopenten-1-yl) undecanoic acid, ( +)-. However, the highest peaks were observed around 38.11 min to 41.2 min. The phytocompounds observed here included akuammilan-17-ol- 10-methoxy 11-(2-cyciopenten-1-yl) undecanoic acid. ( +)-, cyclopentane undecanoic acid methyl ester and cyclopentane undecanoic acid. Significant peaks were also observed at 26.3 and 26.5 min which corresponded to oxirane 2.2″ -(1.4-butanediyl) bis- and cyclopentane undecanoic acid. At 23.05 min, we also see ( +)-2- hydroxy octanoic acid acetate. One more peak of interest was observed at 11.49 min and corresponded to N-nitroso-2-methyl-oxazolidine. Data is shown in Fig. 6 and Table 3.

### Subfraction J

**Fig. 7:**
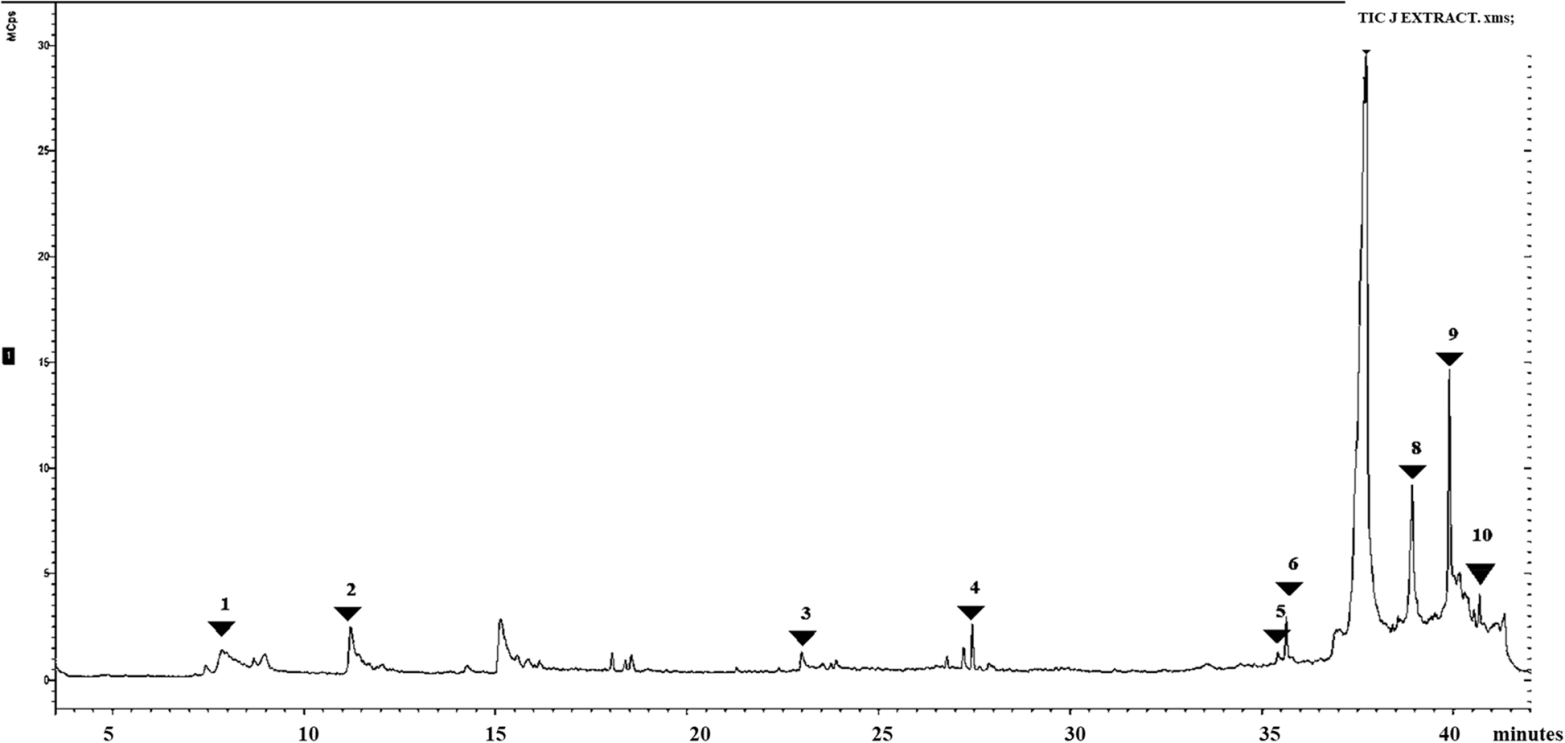
GC–MS chromatogram for sub-fraction J. The numbers indicated in the spectrogram correspond with the peaks of compounds indicated in Table 4 below

Subfraction J had 15 peaks, the peaks of interest were seen at 39.9 min to 41.3 min and corresponded to the following phytoconstituents 1-ethenyl-3 (1-hexenyl) -4- trimethylsilyl cyclopentane, epoxy hexanol and n-decanoic acid. in this subfraction also we see the continued appearance of 11-(2-cyclopenten-1-yl) undecanoic acid at different at 36–37 min. We also see some significant peaks at 11.22 min corresponding to butane nitrile, 2,3-dioxo-, dioxime, O, O" diacetyl-. At 8.9 min propane nitrile, 2-hydroxy- was seen. Data is shown in Fig. 7 and Table 4.

## Discussion

Studies have shown that alpha-glucosidase inhibitors can prevent the development of diabetes in prediabetics at risk of developing diabetes and can also prevent disease progression and the emergence of complications of diabetes [15]. Alpha-glucosidase inhibition has proved to be beneficial not only for postprandial hyperglycemia but also for the management of weight and reduction of insulin resistance [16]. However, the reported gastrointestinal-related adverse effects [13, 15] have highlighted the need for natural alpha-glucosidase inhibitors. Although there was a significant difference in the alpha-glucosidase inhibitory activity of the fruit fractions, the observed bioactivity of *Kigelia* subfractions implies that the phytocompounds of *Kigelia* fruit extract has great potential to prevent the development of diabetes among patients at risk of T2DM and the development of complications of diabetes. The findings in this study also suggest a possible alpha-glucosidase inhibitor that is readily available to Southern African patients considering that conventional alpha-glucosidase inhibitors are not readily available to diabetic patients in most Southern African countries (MOH, 2013; Rossiter,2014). Furthermore, the results of this study suggest an added new natural source of alpha-glucosidase inhibitors that are needed as a safe alternative to existing conventional therapies.

The subfractions of KAFE that demonstrated the best in vitro antidiabetic activity had the indole alkaloids akuammilan-17-ol- 10-methoxy and N-nitroso-2-methyl-oxazolidine. Recent studies have demonstrated the significance that indole alkaloids play in the management of diabetes. Goboza and colleagues reported that they have significant alpha-glucosidase inhibitory activity among other antidiabetic effects [17]. Other studies have shown that oxazolidines have a positive effect on body weight, insulin secretion, and a generally positive outcome in the management of diabetes [18]. Oxirane 2.2″ -(1.4-butanediyl) bis- which contains epoxide was observed. These rings have demonstrated the ability to improve the antidiabetic effects including alpha-glucosidase inhibitory activity of extracts that contain them [19, 20]. Subfraction J, which had the best alpha-glucosidase inhibitory activity also had 1,2-benzanedicarboxylic acid, butyl octyl ester, a naturally occurring phthalic acid ester. These compounds have been reported to possess possible alpha-glucosidase inhibitory activity [21].

We further observed the presence of cyclic fatty acids like 11"(2-cyclopenten-1-yl) undecanoic acid, ( +)- and cyclopentane undecanoic acid. Literature has demonstrated the significant role that fatty acids play in the management of diabetes and other metabolic syndromes as well as their beneficial effect on coronary artery disease [22, 23]. A few studies have demonstrated that some fatty acids like octadecadienoic acid and n-hexadecanoic acid may inhibit alpha-amylase and alpha-glucosidase activity (Go and R, 2019; [24]),however, there is scanty information associating some of the phytocompounds identified in this study to probable antidiabetic activity and particular bioactivity observed in this study. The fatty acid 11"(2-cyclopenten-1-yl) undecanoic acid, ( +)- and cyclopentane undecanoic acid is a cyclic fatty acid that is naturally found in a few plants belonging to the *Hydnocarpus* species and has been associated with its inhibitory activity on *Mycobacterium lepra* [25, 26]. Although there is scanty literature available that highlights its activity in diabetes, we observed the presence of this rare fatty acid across all bioactive subfractions. Other phytocompounds observed in this study have previously been associated with antidiabetic activity. For example, decanoic acid has demonstrated that when it is added to chitosan hydrogels, their antidiabetic activity significantly improves [22]. Moreover, nitrile derivatives were able to enhance the effect of metformin in Rabbit-induced diabetes [27]. The fact that the fruit of Kigelia is crushed together with its seeds could have contributed to the significant presence of fatty acids. This suggests that this fruit and its extracts have the potential to be used as nutraceuticals or pharmaceuticals in the management of metabolic and cardiovascular-related conditions. The fruit would be significant in the lowering of postprandial blood glucose. This study also highlights several compounds that may serve as pharmacophores under natural alpha-glucosidase inhibitors considering the narrow range of alpha-glucosidase inhibitors which are also arrayed with several adverse drug reactions.

## Conclusion

Through activefractions *Kigelia africana* fruit (KAFE) ethyl acetate fraction and its active subfractions rich in fatty acids, indole alkaloids and phenolic compounds have been associated with alpha-glucosidase inhibitory activity. The ethyl acetate fraction had an estimated IC50 10.41 μg/mL. We suggest the use of identified phytocompounds, for the development of lead compounds for inhibition of alpha-glucosidase enzymes and the development of pharmaceutical formulations for cheaper alternative treatments.

## Data Availability

All the data that supports the findings of this study are presented in this manuscript or supplementary information files.
